# Latent class analysis of pregnancy-related anxiety in pregnant women with preterm premature rupture of membranes

**DOI:** 10.3389/fpubh.2026.1921112

**Published:** 2026-10-02

**Authors:** Ruili Zhu, Xiaohui Yan, Lina Zhao

**Affiliations:** Department of Obstetrics and Gynecology, Henan Provincial People’s Hospital, Zhengzhou, China

**Keywords:** influencing factors, latent class analysis, pregnancy-related anxiety, preterm premature rupture of membranes, psychological resilience

## Abstract

**Objective:**

To explore the latent classes of pregnancy-related anxiety and their influencing factors in pregnant women with preterm premature rupture of membranes (PPROM), and to provide evidence for developing targeted psychological interventions.

**Methods:**

Using convenience sampling, 136 pregnant women with PPROM hospitalized in the Department of Obstetrics of Henan Provincial People’s Hospital from January 2023 to March 2025 were enrolled. Surveys were conducted using a general information questionnaire, the Pregnancy-Related Anxiety Questionnaire (PAQ), the Vulnerable Personality Style Questionnaire (VPSQ), and the Connor-Davidson Resilience Scale (CD-RISC). Latent class analysis (LCA) was performed using Mplus 8.3, with the 13 item scores of the PAQ as manifest indicators to determine the optimal number of classes. Univariate analysis and binary logistic regression were used to identify factors influencing class membership.

**Results:**

The mean total PAQ score of PPROM pregnant women was 31.26 ± 6.83. LCA identified two latent classes: the generalized anxiety group (79 cases, 58.1%) showed conditional probabilities >0.6 on all 13 items; the fetal health anxiety group (57 cases, 41.9%) showed conditional probabilities >0.6 only on items 7, 8, and 13 (all belonging to the “worry about fetal health” dimension), whereas the conditional probabilities on the remaining items were generally below 0.6, with items 9 and 10 showing moderate values. Univariate analysis showed statistically significant differences between the two groups in education level, history of adverse pregnancy outcomes, vulnerable personality type, total PAQ score, and resilience score (all *p* < 0.05). Multivariate logistic regression revealed that college degree or above (OR = 0.410, 95%CI: 0.204–0.824) and resilience (OR = 0.924, 95%CI: 0.895–0.954) were protective factors for belonging to the generalized anxiety group, while history of adverse pregnancy outcomes (OR = 2.667, 95%CI: 1.210–5.878) and vulnerable personality (OR = 3.368, 95%CI: 1.696–6.688) were independent risk factors.

**Conclusion:**

Pregnancy-related anxiety in PPROM pregnant women exhibits significant population heterogeneity and can be classified into a generalized anxiety group and a fetal health anxiety group. The two groups differ in the content and focus of anxiety, which suggests that clinical practice should move beyond total-score cut-offs toward content-based assessment. Education level, history of adverse pregnancy outcomes, vulnerable personality, and resilience are independent factors influencing class membership. Graded psychological management based on the content of anxiety may be considered, but the stability and clinical utility of these classes require confirmation in larger, longitudinal studies.

## 1 Introduction

Preterm premature rupture of membranes (PPROM) is defined as spontaneous rupture of the fetal membranes before the onset of labor at <37 weeks of gestation, and represents one of the most common acute obstetric complications (1). Epidemiological studies indicate that the incidence of PPROM ranges from 2.0 to 3.5% of all pregnancies, contributing to approximately 30–40% of preterm births (2). Because the amniotic cavity is exposed to the external environment, PPROM significantly increases the risks of chorioamnionitis, umbilical cord prolapse, placental abruption, and fetal distress; moreover, the earlier the gestational age at rupture, the higher the perinatal mortality rate (3). Currently, there is no definitive curative treatment for PPROM, and most affected women are required to undergo prolonged strict bed rest with elevation of the hips to minimize amniotic fluid leakage while awaiting corticosteroid administration for fetal lung maturation (4).

Pregnancy itself constitutes a major physiological and psychological stressor, and the sudden, unpredictable onset of PPROM further intensifies feelings of fear and loss of control. Pregnancy-related anxiety refers to a specific anxiety experience triggered by pregnancy and peripartum stressors, characterized predominantly by excessive worry about fetal health, delivery outcomes, and adaptation to the maternal role (5). Previous studies have shown that women with high-risk pregnancies exhibit significantly higher levels of pregnancy-related anxiety than those with uncomplicated pregnancies, and persistent anxiety not only reduces treatment adherence but may also affect the intrauterine environment via the hypothalamic–pituitary–adrenal axis, thereby further increasing the risks of preterm birth and adverse neonatal outcomes (6). Therefore, accurate identification of the anxiety characteristics in PPROM women and early intervention are of significant clinical value for improving maternal and neonatal prognosis.

However, most existing studies on anxiety in PPROM women have adopted variable-centered analytical strategies, which rely on comparisons of total anxiety scale scores or regression analyses based on mean values (7). This approach assumes homogeneity of the study population and overlooks potential qualitative differences in anxiety symptom dimensions across individuals. In reality, some women may present with predominant concerns about fetal well-being, others with a primary fear of childbirth, and still others with psychological distress stemming from prolonged bed rest and physical immobility—subtypes of anxiety that cannot be differentiated by a single total score. Latent class analysis (LCA) is a person-centered analytical method that classifies individuals into mutually exclusive, internally homogeneous, and externally heterogeneous latent subgroups based on their response patterns across multiple dimensional items (8). This technique has been applied to investigate psychological heterogeneity in populations such as cancer patients and intensive care unit family members, but its application to pregnancy-related anxiety in PPROM women remains unexplored.

To address this gap, the present study employed latent class analysis to explore the latent classification characteristics of pregnancy-related anxiety in women with PPROM, and to compare differences in demographic and clinical features among the identified classes, with the aim of providing evidence-based support for the development of tiered, individualized psychological intervention strategies by clinical nursing staff.

## 2 Materials and methods

### 2.1 Study participants

Using convenience sampling, we retrospectively reviewed the medical records of pregnant women with PPROM who were hospitalized in the Department of Obstetrics of Henan Provincial People’s Hospital from January 2023 to March 2025. Inclusion criteria: (1) meeting the diagnostic criteria for PPROM as defined in the Guidelines for the Diagnosis and Management of Premature Rupture of Membranes (2015) (9) (positive vaginal pH test (blue color change), positive ferning test, and markedly increased vaginal fluid leakage); (2) singleton pregnancy with gestational age between 28 and 36^+6^ weeks; (3) having completed the routine psychological assessment questionnaires during hospitalization with complete records available for review. Exclusion criteria: (1) presence of severe organic diseases (e.g., heart, liver, kidney) or malignant tumors; (2) confirmed fetal malformations or chromosomal abnormalities; (3) diagnosed psychiatric disorders based on medical record documentation or self-report, or cognitive impairments that would prevent cooperation with the survey.

This retrospective study was approved by the Ethics Review Board of Henan Provincial People’s Hospital (approval No. H2022-012). As the data were derived from anonymized questionnaire records routinely collected during clinical care without additional patient contact, the requirement for written informed consent was waived.

### 2.2 Survey instruments

#### 2.2.1 General information questionnaire

Developed by the researchers based on literature review, this questionnaire collected basic information including maternal age, gravidity, gestational age, education level, average monthly household income per capita, mode of medical expense payment.

#### 2.2.2 Pregnancy-Related Anxiety Questionnaire—(core measurement tool)

The PAQ, developed by Xiao Limin et al. (10), was used. This scale comprises three dimensions—“concern about self,” “worry about fetal health,” and “fear of childbirth”—with a total of 13 items. Each item is rated on a 4-point Likert scale ranging from “no worry” (1 point) to “always worried” (4 points), yielding a total score from 13 to 52, with higher scores indicating greater pregnancy-related anxiety. In this study, the Cronbach’s α coefficient of the scale was 0.86, indicating good internal consistency reliability in this sample. The scores of the 13 items of this scale were used as the manifest indicators for latent class analysis (Table 1).

**Table 1:** Fit indices for latent class models of pregnancy-related anxiety in PPROM women.

Model	K	Log(L)	AIC	BIC	aBIC	Entropy	LMR P	BLRT P	Class probabilities
1	13	−1468.372	2962.744	3005.664	2964.497	—	—	—	1.00
2	27	−1278.915	2611.830	2699.563	2611.918	0.902	<0.001	<0.001	0.58/0.42
3	41	−1242.687	2567.374	2697.256	2565.981	0.813	0.089	<0.001	0.33/0.35/0.32
4	55	−1223.451	2556.902	2728.934	2554.029	0.791	0.124	0.042	0.28/0.30/0.22/0.20

#### 2.2.3 Vulnerable Personality Style Questionnaire—(covariate/influencing factor)

The VPSQ, originally developed by Boyce et al. (11), was used to assess neurotic personality tendencies in pregnant women. It contains 9 items rated on a 5-point Likert scale, with total scores ranging from 9 to 45; a score >15 is considered indicative of a vulnerable personality (11). In this study, the Cronbach’s α coefficient of the VPSQ was 0.78. This scale was used to compare differences in personality traits among different latent anxiety classes.

#### 2.2.4 Connor-Davidson Resilience Scale—(covariate/protective factor)

The CD-RISC, originally developed by Connor and Davidson (12), comprising 25 items across three dimensions (hardiness, strength, and optimism), was used. Each item is scored on a 5-point Likert scale (1–5), with total scores ranging from 25 to 125; higher scores reflect better resilience (12). This scale was used to explore the protective role of resilience across different anxiety classes.

### 2.3 Data collection and quality control

According to the hospital’s clinical protocol, trained ward nurses, as part of standard nursing practice, distributed the paper-based questionnaire battery to patients within 24–48 h after admission (when their emotional state was relatively stable) and informed them that the surveys were used to assess psychological status during hospitalization. Given that patients were required to remain on strict bed rest, questionnaires were uniformly completed independently by the pregnant women themselves. For those unable to write due to intravenous infusion or physical discomfort, the investigator read each item aloud and recorded the woman’s oral responses, with a note indicating “proxy-completed.” All completed questionnaires were filed in the medical record system after discharge. For the present retrospective analysis, we retrieved these existing questionnaire records along with clinical data from the medical records department.

After data extraction, the questionnaires were checked one by one. Questionnaires were excluded if the completion time was too short (<2 min), if more than 10 consecutive items had identical responses, or if >20% of key variables were missing. For questionnaires with occasional missing item-level data within an included scale, the mean of the remaining items in the same subscale was imputed if no more than one item was missing; otherwise the questionnaire was excluded. No missing key data remained in the final analytic sample of 136 participants.

### 2.4 Statistical analysis

SPSS 26.0 was used for data cleaning and descriptive analysis, and Mplus 8.3 was used for latent class analysis (LCA).

The 13 item scores of the PAQ were used as manifest indicators. Each item was dichotomized as follows: responses of 1 or 2 were coded 0, and responses of 3 or 4 were coded 1. Conditional probabilities >0.6 were regarded as indicating a high likelihood of endorsing the anxiety response, whereas conditional probabilities <0.4 were regarded as indicating a low likelihood; these thresholds are commonly used in latent class analysis to facilitate substantive interpretation (13). The analysis started with a 1-class model and progressively increased the number of classes until the optimal model fit was achieved. Model fit evaluation comprehensively considered: the Akaike information criterion (AIC), Bayesian information criterion (BIC), and sample-size adjusted BIC (aBIC), with lower values indicating better fit; an Entropy value >0.8 indicating classification accuracy exceeding 90%; and the statistical significance of adding a class was determined by the *p*-values of the Lo–Mendell–Rubin adjusted likelihood ratio test (LMRT) and the bootstrapped likelihood ratio test (BLRT), with *p* < 0.05 considered statistically significant. The final number of classes was selected based on the combined evidence from model fit indices, entropy, class size, interpretability, and parsimony (13, 14).

Subsequent comparisons: After determining the optimal number of classes, independent-samples t tests were used to compare continuous variables (age, gestational age, PAQ total score, VPSQ score, resilience score) between the two classes, and chi-square tests or Fisher’s exact tests were used for categorical variables. Variables that were significant in univariate analyses were then entered into a multivariate logistic regression (forward stepwise method) to identify independent predictors of latent class membership. The significance level was set at α = 0.05 (two-tailed).

## 3 Results

### 3.1 Baseline characteristics of the study participants

A total of 150 questionnaires were distributed, and 136 valid responses were collected, yielding an effective response rate of 90.7%. The final sample therefore comprised 136 pregnant women with PPROM. The age ranged from 20 to 42 years, with a mean of (30.2 ± 4.8) years; gestational age ranged from 28 to 36^+6^ weeks, with a mean of (33.5 ± 2.1) weeks. There were 58 primiparas (42.6%) and 78 multiparas (57.4%). In terms of education level, 49 women (36.0%) had a college degree or above, while 87 (64.0%) had education below college level. A history of adverse pregnancy outcomes was present in 39 cases (28.7%), and 71 women (52.2%) were classified as having a vulnerable personality. The total PAQ score for the PPROM women was (31.26 ± 6.83), with an average item score of (2.41 ± 0.53).

### 3.2 Latent class analysis results of pregnancy-related anxiety in PPROM women

Using the 13 item scores of the PAQ as manifest indicators, latent class analysis (LCA) was performed on the 136 PPROM women. Starting with a 1-class model and gradually increasing the number of classes, the values of Log(L), AIC, BIC, and aBIC decreased as the number of classes increased. The 2-class model yielded the highest Entropy value (0.902), indicating excellent classification accuracy. Both the LMR and BLRT tests reached significance (both *p* < 0.001), indicating that the 2-class model was significantly superior to the 1-class model. Although the 3-class model showed a slight further decrease in BIC, the LMR test was not significant (*p* = 0.089), entropy decreased to 0.813, and the class structure became less interpretable and less parsimonious. Considering goodness-of-fit, classification quality, and interpretability together, the 2-class solution was ultimately selected as the optimal classification. The average posterior membership probabilities for the two classes were 96.8 and 94.3%, respectively, indicating reliable classification. Using the normal approximation based on the observed proportions, the estimated class proportion was 58.1% (95% CI, 49.8–66.4%) for the generalized anxiety group and 41.9% (95% CI, 33.6–50.2%) for the fetal health anxiety group.

The conditional probability profiles of the 13 PAQ items for the two latent classes are shown in Figure 1.

**Figure 1:**
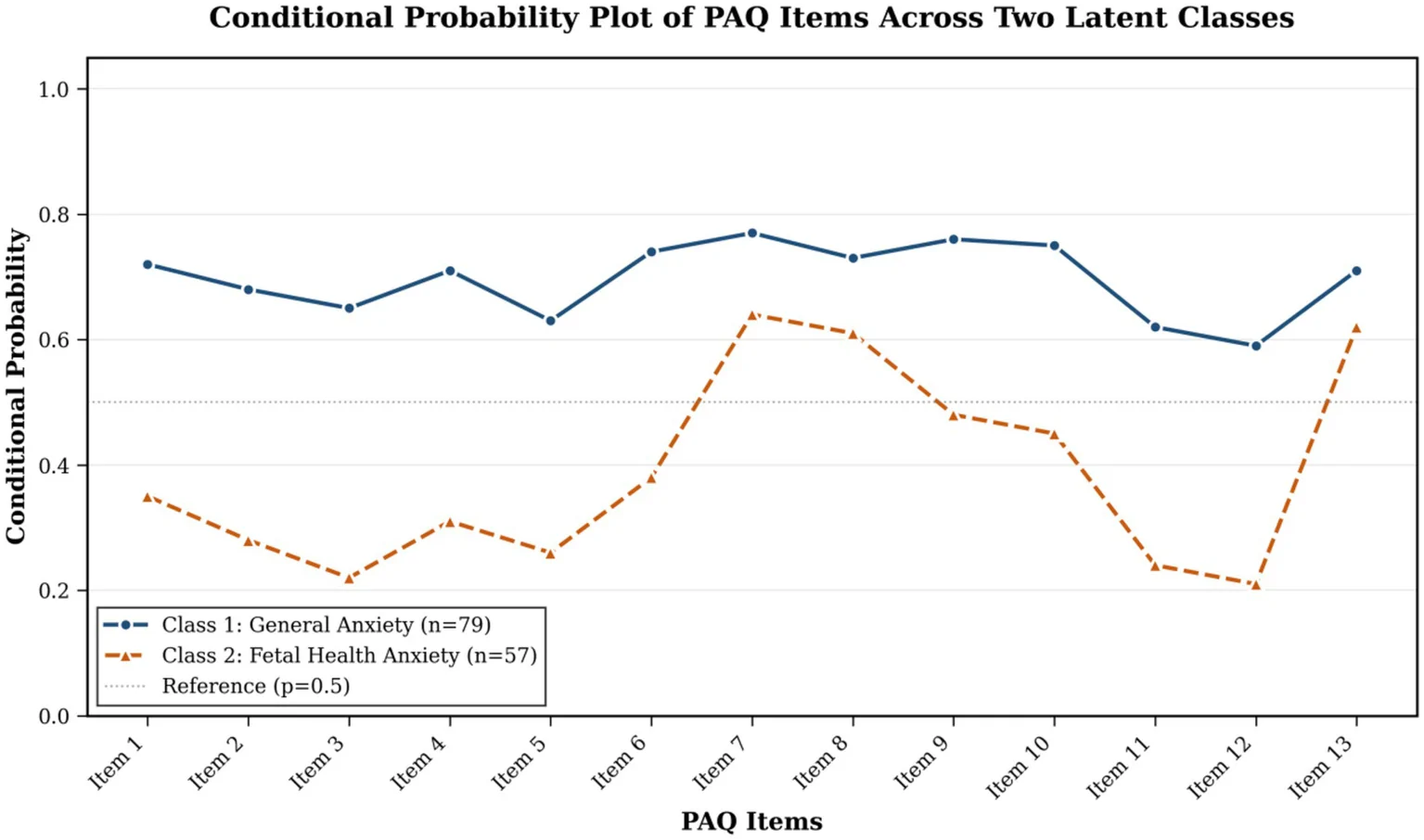
Conditional probability plot of the 13 items on the Pregnancy-related Anxiety Questionnaire (PAQ) across two latent classes among PPROM women. The y-axis represents the conditional probability of endorsing the anxiety response (original score 3 or 4 on each item), and the x-axis represents the 13 PAQ items.

Class 1 (high-anxiety group, *n* = 79, 58.1%) had high conditional probabilities (>0.6) on all 13 PAQ items, indicating pervasive anxiety across various pregnancy-related domains, including concerns about their own physical changes, fetal health, and the delivery process. This class was therefore designated the generalized anxiety group.

Class 2 (low-anxiety with fetal concerns group, *n* = 57, 41.9%) showed high conditional probabilities (>0.6) on three specific items: item 7 (“worry about fetal health”), item 8 (“worry about the child’s intellectual development”), and item 13 (“worry about the negative effects of unhealthy lifestyle on pregnancy”). The conditional probabilities on the remaining items were below 0.6, with items 9 and 10 showing moderate values between 0.4 and 0.6. As these three high-probability items belong to the “worry about fetal health” dimension of the PAQ, this class was designated the fetal health anxiety group. However, the moderate probabilities observed on items 9 and 10 suggest that some concern about general pregnancy outcomes may also be present, although it is less prominent than in the generalized anxiety group.

### 3.3 Comparison of characteristics between different latent classes of PPROM women

Univariate analyses comparing the generalized anxiety group and the fetal health anxiety group showed statistically significant differences in education level, history of adverse pregnancy outcomes, vulnerable personality type, total PAQ score, and resilience score (all *p* < 0.05). No statistically significant differences were found in age, gestational age, or parity type (all *p* > 0.05). See Table 2.

**Table 2:** Comparison of characteristics between different latent anxiety classes in PPROM women.

Variable	Generalized anxiety group (*n* = 79)	Fetal health anxiety group (*n* = 57)	t/χ^2^	*p*
Age (years, mean ± SD)	30.5 ± 4.9	29.8 ± 4.6	0.844	0.400
Gestational age (weeks, mean ± SD)	33.2 ± 2.2	33.9 ± 1.9	−1.937	0.055
Education level [n (%)]			3.891	0.049
Below college	56 (70.9)	31 (54.4)		
College or above	23 (29.1)	26 (45.6)		
Parity type [n (%)]			0.024	0.876
Primipara	34 (43.0)	24 (42.1)		
Multipara	45 (57.0)	33 (57.9)		
History of adverse pregnancy outcomes [n (%)]			4.182	0.041
Yes	28 (35.4)	11 (19.3)		
No	51 (64.6)	46 (80.7)		
Vulnerable personality [n (%)]			11.486	0.001
Yes	51 (64.6)	20 (35.1)		
No	28 (35.4)	37 (64.9)		
PAQ total score (mean ± SD)	36.52 ± 4.28	24.01 ± 3.95	17.486	<0.001
Resilience (CD-RISC, mean ± SD)	62.37 ± 12.54	73.86 ± 10.91	−5.536	<0.001

Pearson chi-square tests were used for categorical variables.

### 3.4 Multivariate logistic regression analysis of factors influencing latent class membership in PPROM women

Using latent class membership as the dependent variable (generalized anxiety group = 1, fetal health anxiety group = 0), the variables that were significant in univariate analyses (education level, history of adverse pregnancy outcomes, vulnerable personality type, and resilience score) were entered as independent variables into a binary logistic regression with forward stepwise selection. Variable assignments are shown in Table 3.

**Table 3:** Assignment of independent variables.

Variable	Assignment
Education level	College or above = 1, below college = 0
History of adverse pregnancy outcomes	Yes = 1, No = 0
Vulnerable personality type	Vulnerable = 1, Non-vulnerable = 0
Resilience score	Original value entered

The multivariate logistic regression results showed that a college degree or above (OR = 0.410, 95% CI: 0.204–0.824) was a protective factor for belonging to the generalized anxiety group (i.e., women with higher education were more likely to belong to the fetal health anxiety group). A history of adverse pregnancy outcomes (OR = 2.667, 95% CI: 1.210–5.878) and vulnerable personality (OR = 3.368, 95% CI: 1.696–6.688) were independent risk factors for belonging to the generalized anxiety group. Higher resilience scores (OR = 0.924, 95% CI: 0.895–0.954) were associated with a lower risk of being in the generalized anxiety group, indicating resilience is a protective factor against pregnancy-related anxiety (Table 4).

**Table 4:** Multivariate logistic regression analysis of factors influencing latent class membership in PPROM women.

Independent variable	β	SE	Wald χ^2^	OR	95% CI	*p*
Education (college or above)	−0.892	0.356	6.278	0.410	0.204–0.824	0.012
Adverse pregnancy history (yes)	0.981	0.403	5.926	2.667	1.210–5.878	0.015
Vulnerable personality (yes)	1.214	0.350	12.034	3.368	1.696–6.688	0.001
Resilience score	−0.079	0.016	24.391	0.924	0.895–0.954	<0.001

## 4 Discussion

As a common acute obstetric complication, preterm premature rupture of membranes (PPROM) imposes tremendous psychological stress on pregnant women due to its sudden onset and direct threat to maternal and fetal safety. However, for a long time, clinical understanding of anxiety in PPROM women has remained at a coarse level of “overall elevated” scores, neglecting the qualitative differences in the content and intensity of anxiety across individuals. This study is among the first to apply latent class analysis to pregnancy-related anxiety in PPROM women, and identified two distinct latent subgroups—the generalized anxiety group and the fetal health anxiety group—confirming significant heterogeneity in anxiety experiences. This finding has important methodological implications: relying solely on the total PAQ score may reflect overall severity, but it fails to reveal substantive differences in the focus of anxiety content across individuals, and this difference is precisely the prerequisite for developing precise intervention strategies.

From the conditional probability profiles of the two classes, the distinction is not simply a matter of “high anxiety” versus “low anxiety,” but rather reflects clear qualitative differences. The generalized anxiety group, accounting for 58.1%, showed conditional probabilities >0.6 on all 13 PAQ items, indicating a diffuse pattern of anxiety—these women were not only worried about fetal well-being but also highly concerned about their own physical changes, safety during delivery, and the availability of partner support. In contrast, the fetal health anxiety group showed high probability anxiety mainly on items 7, 8, and 13, all of which belong to the “worry about fetal health” dimension. The moderate probabilities on items 9 and 10 suggest that some broader pregnancy concerns are present, but they are considerably less prominent than in the generalized anxiety group. This pattern is consistent with the theoretical core of “pregnancy-specific anxiety” proposed by Matthey et al. (15), which emphasizes that the primary activation in high-risk pregnancy states is fetal-directed worry driven by maternal protective motivation, rather than the typical manifestations of generalized anxiety disorder. For clinical nursing, this implies that for women in the fetal health anxiety group, comprehensive anxiety intervention may not be necessary; providing information support focused on fetal health and communicating medical uncertainty may be sufficient. Conversely, for the generalized anxiety group, multidimensional and multidisciplinary psychological intervention may be required. However, because this study was cross-sectional and assessed anxiety only during the acute admission period, it remains unclear whether these two classes represent stable psychological subtypes or temporary response patterns to hospitalization. This distinction should be tested in future longitudinal studies.

This study also found that the total PAQ score of PPROM women was (31.26 ± 6.83), with an average item score of (2.41 ± 0.53), which was higher than the level reported in the original validation study of the PAQ in a general pregnant population (10). This difference is not surprising—PPROM women face a dense array of intense stressors: the risk of preterm delivery, neonatal complications, strict bed rest restrictions, and immediate threats of infection and cord prolapse, all of which naturally drive anxiety levels upward. However, our LCA results further revealed that this “overall elevated” mean value masks substantial internal differentiation—the generalized anxiety group had an average item score approximately 2.81, whereas the fetal health anxiety group scored approximately 1.85, the latter falling within the range reported for uncomplicated pregnant women (10). This finding poses a direct challenge to current clinical assessment strategies. At present, most obstetric units still use a fixed threshold of the total scale score for psychological evaluation of PPROM women. This “one-size-fits-all” cut-off strategy may lead to two types of bias: on the one hand, misclassifying women in the fetal health anxiety group as “high anxiety” and imposing unnecessary interventions, thereby wasting scarce clinical psychological resources; on the other hand, missing severe psychological distress in some women in the generalized anxiety group whose total scores do not reach the threshold. The classification framework provided by this study suggests that when PAQ scores are predominantly concentrated on items within the “worry about fetal health” dimension, clinicians may prioritize targeted information support such as fetal monitoring results and bedside ultrasound interpretations; however, when anxiety spreads across multiple dimensions, referral to psychological specialists or introduction of structured psychological interventions should be considered. Nevertheless, because the fetal health anxiety group may partly reflect an adaptive and expected concern in the context of PPROM, clinicians should avoid pathologizing such focused worry. Future studies should compare these classes against clinical diagnostic interviews and birth outcomes to clarify whether the fetal health anxiety group represents a clinically significant syndrome or a normal response to a high-risk pregnancy.

In the multivariate analysis, the protective effect of education level on latent class membership deserves attention. This study found that the risk of belonging to the generalized anxiety group for women with a college degree or above was only 0.410 times that of those with education below college level. Similar findings have been reported in previous studies on high-risk pregnancies (16–18), and the underlying mechanisms may involve multiple aspects. First, women with higher education generally possess stronger abilities to access and appraise health information, and they tend to have a more rational and structured cognitive framework regarding the etiology, treatment pathways, and neonatal outcomes of PPROM, which can effectively curb catastrophic thinking about unknown risks. Second, individuals with higher educational attainment often develop more mature problem-focused coping strategies—actively consulting, seeking reliable information, and effectively communicating with healthcare providers—rather than indulging in ruminative thoughts (19). Third, the positive association between education level and health literacy enables highly educated women to better understand the evidence-based rationale behind medical instructions, thereby gaining a greater sense of control during passive constraints such as bed rest and activity restrictions. From a clinical perspective, for PPROM women with low education, nursing staff should consider using structured teach-back methods to assess understanding and address misconceptions, rather than relying only on standard written educational materials.

A history of adverse pregnancy outcomes was another statistically significant risk factor. PPROM women with such a history had a 2.667-fold higher risk of belonging to the generalized anxiety group compared to those without. This association can be understood from a traumatic psychology perspective. Previous adverse pregnancy outcomes may constitute an unresolved pregnancy-related trauma; when the current pregnancy is again complicated by PPROM, traumatic memories are immediately activated, and the woman faces not only the real threat of current membrane rupture but also the anticipatory fear of “history repeating itself” (20, 21). This dual temporal orientation of anxiety may make it difficult to contain worry within specific domains, leading to the diffuse characteristics of the generalized anxiety group. It is worth noting that in the fetal health anxiety group, 19.3% of women also had a history of adverse pregnancy outcomes, indicating that not all women with traumatic experiences develop generalized anxiety. This discrepancy suggests that the effect of adverse pregnancy history on anxiety may be moderated by cognitive processing styles—if a woman can integrate her previous experience into a narrative of “although unfortunate, it is in the past, and this pregnancy is different,” her anxiety may remain more focused and less severe. This hypothesis should be examined in future research.

Among all factors included in the analysis, vulnerable personality had the largest effect size—PPROM women with a vulnerable personality had a 3.368-fold higher risk of belonging to the generalized anxiety group compared to those without. This finding is consistent with previous studies identifying neuroticism/vulnerable personality as a stable personality predictor of pregnancy-specific anxiety (22, 23). From a neurobiological perspective, individuals with high vulnerability may exhibit excessive amygdala reactivity to threatening stimuli and insufficient prefrontal cortical regulation of emotions (24), rendering it difficult to confine anxiety within specific cognitive domains when facing a highly uncertain stressor like PPROM, instead rapidly generalizing into diffuse hypervigilance. However, personality traits are not deterministic; even among women with vulnerable personality, 35.1% still belonged to the fetal health anxiety group, which is narrower in range and milder in severity. This suggests that the influence of personality can be buffered by other factors, such as resilience and adequate information support. In clinical practice, PPROM women who screen positive on the VPSQ should be identified as high-risk for generalized anxiety and receive more frequent psychological attention in the early admission period, while avoiding labeling prejudices and instead dynamically assessing changes in actual anxiety content and severity.

Psychological resilience emerged as another important protective factor in this study. For each 1-point increase in the CD-RISC score, the risk of belonging to the generalized anxiety group decreased by approximately 7.6%, and this protective effect remained independent after controlling for personality, education, and other factors. The CD-RISC score in the generalized anxiety group (62.37 ± 12.54) was significantly lower than that in the fetal health anxiety group (73.86 ± 10.91), with a difference of 11.49 points, representing a medium-to-large effect size. The protective mechanism of resilience can be understood through two pathways. The first is the cognitive reappraisal pathway—when facing physical discomforts such as bed rest and amniotic fluid leakage, individuals with high resilience tend to interpret these experiences as “necessary costs to ensure the baby’s safety” rather than “frightening signals of bodily loss of control,” and this cognitive reframing effectively limits the generalization of anxiety. The second is the behavioral activation pathway—even under conditions of severely restricted physical activity, individuals with high resilience still actively seek meaning and a sense of control, such as consistently recording fetal movement diaries or participating in care decisions. Resilience is not an exclusive trait but a psychological resource that can be enhanced through training (25). Brief resilience training programs have shown benefits for anxiety and depressive symptoms in clinical populations (25), and the present findings provide a rationale for examining such interventions in PPROM women, particularly those with both low resilience and low education.

### 4.1 Limitations

This study has several limitations. First, the cross-sectional design precludes causal inference between anxiety classes and influencing factors; resilience may be a stable trait shaped by previous experiences or may be depleted under current stress, and bidirectional relationships await elucidation through longitudinal follow-up. Second, the sample size was relatively small (*n* = 136). Although this met the basic requirements for LCA with 13 indicators (13, 14), it may have limited the ability to detect a more refined three-or four-class solution. Formal power analysis for LCA was not conducted because statistical power depends on unknown class separation and indicator prevalence; therefore, replication in larger multicenter samples is needed to confirm the two-class structure and to examine whether additional clinically meaningful subgroups exist. Third, the PAQ was administered only once, within 24–48 h after admission, and the acute stress of hospitalization may have influenced the response patterns; the identified classes therefore represent anxiety patterns during the acute stage of PPROM and should not be interpreted as stable psychological subtypes. Fourth, pregnancy complications such as gestational hypertension and diabetes mellitus were not systematically assessed in this study, and their potential confounding effects on anxiety class membership could not be evaluated. Fifth, sensitivity analyses using alternative thresholds for conditional probability or the original four-point ordinal response format were not conducted; such analyses would further support the robustness of the classification. Finally, the use of convenience sampling from a single hospital may have introduced selection bias and limited generalizability.

## 5 Conclusion

This study, through latent class analysis, confirmed that pregnancy-related anxiety in PPROM women exhibits significant population heterogeneity and can be classified into a generalized anxiety group and a fetal health anxiety group. The two groups differ in the content and focus of anxiety, suggesting that clinical assessment should incorporate content-based identification rather than relying solely on total-score cut-offs. Education level, history of adverse pregnancy outcomes, vulnerable personality, and resilience are independent predictors of class membership, with vulnerable personality showing the strongest risk effect and resilience showing a stable protective effect. However, the cross-sectional design and modest sample size mean that these classes should be regarded as preliminary and not as confirmed psychological subtypes. Future research should use longitudinal designs, larger samples, and formal replication to verify the stability of the classification and to evaluate whether classification-guided interventions can improve psychological outcomes in this high-risk obstetric population.

## Data Availability

The raw data supporting the conclusions of this article will be made available by the authors, without undue reservation.
